# Taking AIM at Influenza: The Role of the AIM2 Inflammasome

**DOI:** 10.3390/v16101535

**Published:** 2024-09-27

**Authors:** Dianne W. Xu, Michelle D. Tate

**Affiliations:** 1 Center for Innate Immunity and Infectious Diseases, Hudson Institute of Medical Research, Clayton, VIC 3168, Australia; 2 Department of Molecular and Translational Sciences, Monash University, Clayton, VIC 3168, Australia

**Keywords:** AIM2 inflammasome, influenza A virus, DNA sensing, cytosolic DNA, inflammation, immunotherapy

## Abstract

Influenza A viruses (IAV) are dynamic and highly mutable respiratory pathogens that present persistent public health challenges. Inflammasomes, as components of the innate immune system, play a crucial role in the early detection and response to infections. They react to viral pathogens by triggering inflammation to promote immune defences and initiate repair mechanisms. While a strong response is necessary for early viral control, overactivation of inflammasomes can precipitate harmful hyperinflammatory responses, a defining characteristic observed during severe influenza infections. The Absent in Melanoma 2 (AIM2) inflammasome, traditionally recognised for its role as a DNA sensor, has recently been implicated in the response to RNA viruses, like IAV. Paradoxically, AIM2 deficiency has been linked to both enhanced and reduced vulnerability to IAV infection. This review synthesises the current understanding of AIM2 inflammasome activation during IAV and explores its clinical implications. Understanding the nuances of AIM2’s involvement could unveil novel therapeutic avenues for mitigating severe influenza outcomes.

## 1. Introduction

Influenza viruses of the *Orthomyxoviridae* family are significant contributors to seasonal epidemics and sporadic pandemics, impacting millions worldwide. The World Health Organization estimates that seasonal influenza leads to 3–5 million cases of severe illness annually, with respiratory-related deaths reaching up to 650,000 [1]. Notably, influenza A virus (IAV) is notorious for its ability to rapidly evolve through antigenic drift and shift mechanisms and its zoonotic potential to cross-infect a diverse range of hosts, including humans, birds, and swine [2,3,4]. Of note, avian IAVs circulating in wild birds, pose a major pandemic threat. Historical IAV pandemics over the last century, such as the 1918 ‘Spanish flu’, the 1957 ‘Asian flu’, and the 1968 ‘Hong Kong flu’ caused an estimated cumulative of more than 50 million deaths globally [5,6]. More recently, the 2009 H1N1 pandemic, often referred to as ‘swine flu’, led to an estimated 151,700–575,400 deaths during its initial year of circulation [7]. These exemplify the catastrophic potential of emergent IAV strains.

Clinical presentation of IAV in humans varies from often mild, self-limited infections colonised to the upper respiratory tract to lethal infections of the lower respiratory tract. Severe disease is typically characterised by an overly amplified inflammatory response, defined by the elevated release of pro-inflammatory cytokines, often referred to as a “cytokine storm” [8,9,10]. This leads to substantial tissue damage, lung pneumonia, and the development of acute respiratory distress syndrome (ARDS), which is often fatal [11,12]. The innate immune system is the first line of defence against invading pathogens; however, excessive or uncontrolled responses play a crucial role in the pathogenesis of severe IAV [9,13]. Given the limitations of current IAV vaccines, which require frequent updates to match predicted circulating strains and the paucity of effective anti-influenza drugs, a deeper understanding of innate immune mechanisms is essential to develop host-directed therapeutic strategies that offer broad-spectrum application [3,14]. This is especially pertinent in the face of evolving novel strains with pandemic potential.

### Innate Immune Response to Infection

Upon infection of resident alveolar macrophages and respiratory epithelial and endothelial cells, conserved microbial motifs known as pathogen-associated molecular patterns (PAMPs), as well as damage-associated molecular patterns (DAMPs) such as endogenous alarmins released from damaged and dying cells. PAMPs and DAMPs are recognised by germline-encoded sensors termed pattern recognition receptors (PRRs) [15]. Broadly, PRRs can be classified into five key innate sensing categories that recognise viral components: toll-like receptors (TLRs), retinoic acid-inducible gene-I (RIG-1)-like receptors (RLRs), nucleotide and oligomerisation domain, leucine-rich repeats-containing proteins (NLRs), absent in melanoma (AIM2)-like receptors (ALRs), and C-type lectin receptors (CLRs).

In the context of IAV infection, overwhelming evidence has demonstrated that TLR3 and TLR7 detect double-stranded RNA (dsRNA) and single-stranded RNA (ssRNA) in endosomes, while RIG-I recognises cytosolic 5′triphosphate ssRNA generated as a result of viral replication [15,16,17,18,19]. Additionally, the NLR family pyrin domain containing 3 (NLRP3) is activated by both PAMPs and DAMPs, such as reactive oxygen species (ROS), changes in ionic gradients and trans-Golgi membrane integrity, extracellular adenosine triphosphate (ATP), and influenza viral proteins such as PB1-F2 [20,21,22,23,24,25].

Upon activation of NLRP3, apoptosis-associated speck-like proteins (ASC) and caspase-1 are recruited to oligomerise and form a multi-protein complex termed the ‘inflammasome’ [8,26]. In its active form, the inflammasome is responsible for the proteolytic processing of pro-caspase-1, leading to its cleavage and subsequent release of IL-1 family cytokines (e.g., pro-IL-1β → IL-1β, pro-IL-18 → IL-18), which are among the most potent inflammatory mediators in the body [27]. Genetic deficiency or therapeutic inhibition of NLRP3 in mice has been shown to result in greater resistance to H3N2 and H7N9 infections [28,29,30]. Excessive IL-1β and IL-18 have been associated with disease severity and poor prognosis during seasonal H1N1, H7N9, and H3N2 infections [28,29]. Regardless of the infection stage in H1N1 and H3N2 infected mice, anti-IL-1β therapy reduced cellular infiltration and lung inflammation in the bronchoalveolar lavage fluid (BALF) and improved survival [31]. In addition to these cytokines, caspase-1 also cleaves and activates gasdermin D, a pore-forming protein that induces a lytic and inflammatory form of cell death called pyroptosis [32,33,34]. Mice deficient in gasdermin D have been shown to be protected against IAV-induced hyperinflammation and lung damage [35].

**Figure 1:**
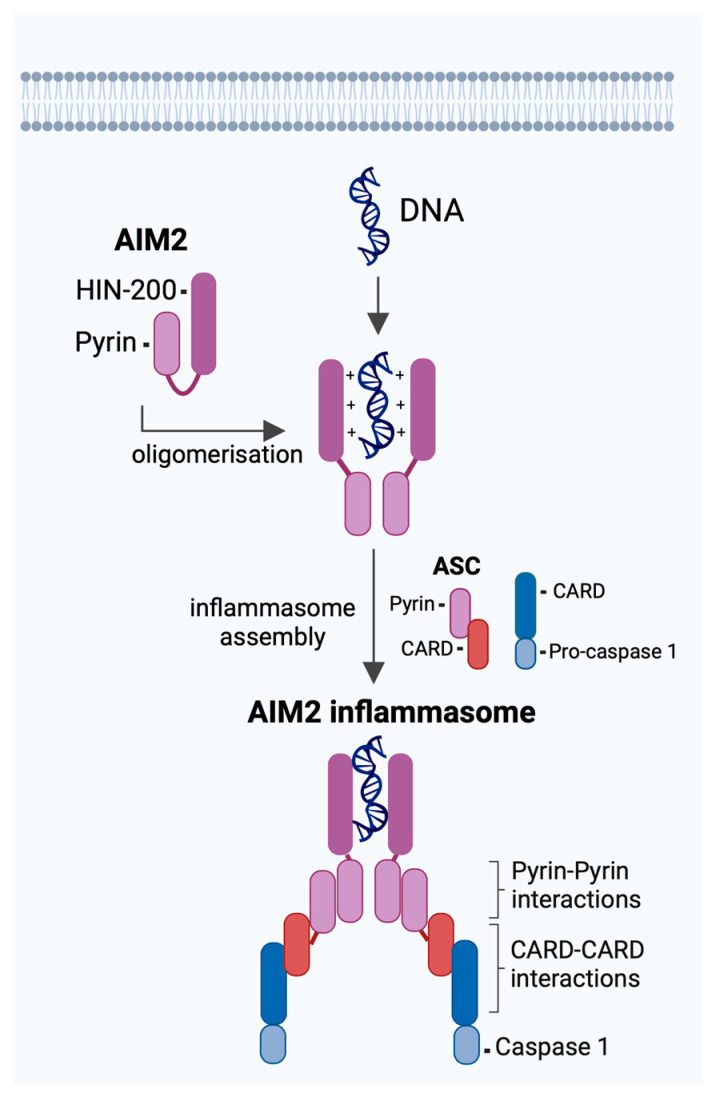
Molecular mechanism of Absent in melanoma 2 (AIM2) inflammasome activation. DNA sensor, AIM2, is structurally composed of two functional domains: the C-terminal hematopoietic interferon-inducible nuclear proteins with a 200 amino acid repeat (HIN-200) domain and the N-terminal Pyrin domain. In the absence of DNA, AIM2 resides in an autoinhibited state through intramolecular interactions between the HIN-200 and Pyrin domains. Upon recognition of cytosolic viral or host DNA, the HIN-200 domain directly binds to the sugar-phosphate backbone of dsDNA in a sequence-independent manner. This interaction results in the recruitment of an inflammasome adaptor, apoptosis-associated speck-like protein containing a CARD (ASC), which interacts via pyrin domain homotypic interactions with AIM2. ASC further recruits pro-caspase-1 through CARD–CARD interactions, leading to the assembly of a multi-protein signalling complex termed the AIM2 inflammasome.

In addition to the NLRP3 inflammasome, the AIM2 inflammasome—a detector of cytosolic double-stranded DNA (dsDNA)—has unexpectedly emerged as a sensor for IAV [36,37]. Unlike NLRP3, which responds to RNA viruses by indirectly detecting viral RNA, cellular stress and damage, AIM2 directly binds to cellular or viral dsDNA released during infection or cell death [26,38] (Figure 1). This sensing leads to apoptosis-associated speck-like protein containing a CARD (ASC) oligomerisation, the recruitment of pro-caspase-1 to the inflammasome complex, and the subsequent release of IL-1β and IL-18. AIM2 inflammasome activation and increased cell-free DNA (cfDNA) levels have been observed in coronavirus, another respiratory RNA virus, where it is positively associated with worsened respiratory symptoms and lung fibrosis [39,40,41,42]. This suggests that the AIM2 inflammasome may also be a valuable therapeutic target in addressing excessive inflammation observed in severe IAV infections. Over the past decade, significant advancements have been made in understanding the role of inflammasomes during IAV infection, revealing their dual role in host defence and immunopathology [8,43]. This review will discuss the recent progress in our understanding of how the AIM2 inflammasome modulates viral infections. We focus specifically on respiratory RNA viruses and their functional orchestration in inducing AIM2 cascades during severe hyperinflammatory IAV infections. We highlight the potential role of targeting AIM2 in the immunotherapeutic landscape.

## 2. AIM2 Inflammasome in Response to Viral Infections

AIM2, a member of the hematopoietic interferon (IFN)-inducible nuclear proteins with a 200 amino acid repeat (HIN-200) family, is comprised of two distinct structural domains: the C-terminal HIN-200 domain and an N-terminal pyrin (PYD) domain [26]. Fifteen years ago, four seminal studies independently identified AIM2 as a cytosolic dsDNA sensor. Three key characteristics underpinning AIM2-DNA interactions were elucidated: (i) AIM2 preferentially binds dsDNA and GC-rich sequences, (ii) dsDNA detection is independent of nucleotide sequence specificity; however, optimal inflammation activation requires at least 80 base pairs, and (iii) the C-terminal HIN-200 domain binds to dsDNA, while the N-terminal PYD domain recruits ASC to activate caspase-1 [38,44,45,46]. In the absence of cytosolic bacterial, viral, or host DNA, the HIN-200 domain engages with the PYD domain to prevent autoactivation [47]. Successful DNA binding relives the PYD domain to recruit ASC and caspase-1 to form the AIM2 inflammasome, a process that occurs autonomously of other inflammasomes like NLRP3 (Figure 1) [47,48].

### 2.1. DNA Viruses

Current evidence underscores the pivotal role of the AIM2 inflammasome in detecting DNA viruses. In AIM2 deficient mice (*Aim2*^−/−^) infected with vaccinia virus (VACV) and mouse cytomegalovirus (MCMV), reduced caspase-1 activation and decreased secretion of IL-1β and IL-18 were observed, suggesting AIM2-dependent induction of pro-inflammatory cytokines in a pyroptotic-driven manner [49]. Elevated levels of AIM2 have been reported in monocytes infected with Epstein–Barr virus (EBV) and in keratocytes infected with human papillomavirus (HPV) [50,51]. On the contrary, herpes simplex virus 1 (HSV1) appears to be preferentially sensed by NLRP3 rather than AIM2 [48,49]. Indeed, previous findings suggest HSV1 viral protein, VP22, inhibits AIM2 activation, preventing IL-1β secretion, which ultimately facilitates viral replication [52,53]. There is an incomplete understanding of how DNA viruses modulate AIM2 inflammasome activation, and further research is necessary to identify the therapeutic potential of AIM2 in DNA viruses.

### 2.2. RNA Viruses

While the recognition of DNA and RNA viruses has traditionally been attributed to distinct receptors, accumulating evidence suggests this dichotomy is not as rigid as previously thought. RNA sensor RIG-I, for example, has been implicated in indirectly detecting DNA virus HSV1, challenging the established paradigm of strict RNA- and DNA-virus recognition by PRRs [54,55,56]. Similarly, DNA sensors have been found to respond to RNA viruses. IFNs, key antiviral cytokines produced in response to RNA and DNA viruses, are part of this cross-sensing response, and AIM2 has now been well-described as an IFN-stimulated gene (ISG) [57]. Infections caused by ssRNA viruses, Chikungunya (CHIKV), Zika (ZIKV), and West Nile (WNV), have been observed to upregulate AIM2 expression in dermal fibroblasts [58,59,60]. Similar findings were reported in Enterovirus A71 (EV71) infection in human neuroblastoma cells [61]. This study found that in vitro genetic inhibition of AIM2 via siRNA reduced pyroptosis, leading to a significant increase in viral infection suggesting AIM2-mediated pyroptosis is beneficial during EV71 infection. To date, there is no evidence in the literature to indicate RNA directly binds to AIM2. The precise mechanisms by which RNA viruses trigger AIM2 activation remain to be elucidated. Since no dsDNA is produced during IAV replication, it is hypothesised that the release of dsDNA from damaged host cells, such as mitochondrial (mtDNA) or nuclear (nDNA) DNA, may be ligands for AIM2. During pyroptotic cell death mediated by gasdermin D and possibly gasdermin E, the release of cellular contents, including DNA, could have the potential to promote AIM2 activation. Further, the role of AIM2 during RNA viral infection, whether beneficial or detrimental, is still largely undetermined. Nonetheless, the recent discovery that AIM2 can also be activated by RNA viruses reveals a broader functional repertoire of viral recognition.

## 3. AIM2 Inflammasome Is Activated during IAV Infection

The role of the AIM2 inflammasome in IAV infection is a topic of active investigation, with emerging evidence highlighting its dualistic role in both protective and pathogenic outcomes (summarised in Table 1) [36,37]. Genetic evidence from a 2016 study provided the first insights implicating AIM2’s involvement in IAV-induced inflammation. Specifically, *Aim2*^−/−^ mice, but not their wild-type (WT) counterparts, exhibited heightened cellular lung infiltration and markedly lower survival rates following a lethal challenge of the mouse-adapted H1N1 strain A/PR/8/34 (PR8) [37]. While a transient reduction in IL-1β, IL-6 and tumour necrosis factor (TNF) secretion was observed during acute infection, levels returned back to WT or higher 5 days post-infection (dpi), suggesting ablation of AIM2 may rather facilitate severe inflammation [37]. Mechanistically, this activation of AIM2 was linked to the release of damaged cellular debris accumulating in the lungs. Indeed, Li and colleagues demonstrated that IAV infection induces significant DNA damage by diminishing the expression of DNA damage repair proteins, which persists past viral clearance [62].

A subsequent study in 2017 confirmed IAV infection-induced dsDNA release as an activator of AIM2 and established its functional activation of the AIM2 inflammasome complex. Interestingly, these experiments revealed that AIM2 exacerbates the IAV immune response, characterised by elevated secretion of bioactive caspase-1 and IL-1β in alveolar macrophages and human primary alveolar type II cells, which ultimately promoted pyroptosis and increased mortality in murine models of PR8 and A/Ca/07/09 (CA07) H1N1 infection [36]. Moreover, IAV-induced increases in lactate dehydrogenase (LDH), albumin, TNF, and immune cell infiltration were significantly reduced in *Aim2*^−/−^ mice, correlating with attenuated lung damage [36]. While the virus had been effectively cleared 9 dpi, Zhang and team found significant lung damage in WT but not *Aim2*^−/−^ mice following. Considering that lung damage and inflammation were not assessed past day 3 by Schattgen, but DNA damage and release continue past 6 dpi, it may be conceivable that AIM2 has a temporal role during IAV, being protective early and harmful later during disease as recently observed with NLRP3 [28]. Collectively, these findings establish cytoplasmic host DNA generated during IAV infection as a key activator of AIM2, advancing our understanding of innate immune recognition mechanisms. The divergent conclusions drawn by these studies may stem from inconsistencies in mouse genetics, viral strains, dose, and/or propagation methods (Table 1). The highly virulent, mouse-adapted PR8 H1N1 strain has been shown to induce high mortality at 50 PFU in C57BL/6J mice [28,63]. It is possible that the 4000 PFU used by Zhang et al., compared to the 40,000 PFU employed by Schattgen et al., may elicit different host immune responses. While no direct comparison exists between PR8 PFU and inflammasome activation, dose-dependent immune responses to PR8 have been reported, suggesting that different doses could lead to varied AIM2 activation [64]. Differences in PR8 strains used and inoculum volume may also affect infection outcomes and require better standardisation. A study has demonstrated that mice recovered faster following a lethal PR8 dose when a smaller inoculum volume was used (25 µL compared to 50 µL) [65]. Future research should aim to clarify the impact of these variables on AIM2 activation to provide a deeper understanding of its role in mediating IAV-induced immune responses.

**Table 1:** Comparative analysis of studies assessing Absent in Melanoma 2 (AIM2) activation during Influenza A virus (IAV) infection.

Methodological Aspect	Studies/Year		
	Schattgen et al. (2016) [37]	Zhang et al. (2017) [36]	Moriyama et al. (2020) [66]
Key influenza infection findings	Transient reduction of IL-1β, IL-6, and TNF secretion in lung homogenates from *Aim2*^−/−^ mice at 3 dpi. Increased albumin in BALF 3 dpi, and CD4^+^, CD8^+^ T cells, and immature macrophages in the lung of *Aim2*^−/−^ mice at 5 dpi. *Aim2*^−/−^ mice displayed reduced survival.	siRNA knockout of AIM2 reduced IL-1β, TNF, and CCL5 secretion from primary human macrophages but not epithelial cells. Reduced pro- and cleaved-forms of caspase-1 and IL-1β in lung tissue and BALF 3 dpi of *Aim2*^−/−^ mice. Reduced LDH, albumin, TNF, and infiltration of inflammatory cells in the lung of *Aim2*^−/−^ mice at 3 dpi. *Aim2*^−/−^ mice displayed improved survival and reduced lung damage 9 dpi.	Reduced IL-1β secretion in *Aim2*^−/−^ BMDMs.
Study model	C57BL/6 mice	Human alveolar macrophages and alveolar epithelial type II cells C57BL/6J mice	Murine primary BMDMs
Animal source	*Aim2*^−/−^ mice generated in-house and backcrossed to C57BL/6 mice.	*Aim2*^−/−^ mice (The Jackson Laboratory) backcrossed to C57BL/6J (The Jackson Laboratory)	N/A
Virus Strain	A/Puerto Rico/8/34 H1N1	A/Puerto Rico/8/34 H1N1 A/California/07/09 H1N1	A/Puerto Rico/8/34 H1N1
Virus Preparation	Embryonated chicken eggs	Madin-Darby Canine Kidney cells	Embryonated chicken eggs
Viral Dose	Mice: 40,000 PFU in 30 µL PBS	Cells: MOI of 1 Mice: 40/4000 PFU (A/PR/8/34) or 5000/500,000 PFU (A/CA/07/09) in 50 µL DMEM	Cells: MOI of 3–10
In vivo route of infection	Intranasal	Intranasal and intratracheal	N/A

Interleukin (IL), tumour necrosis factor (TNF), days post-infection (dpi), plaque-forming units (PFU), bone-marrow-derived-macrophages (BMDMs), bronchoalveolar lavage fluid (BALF), lactate dehydrogenase (LDH), multiplicity of infection (MOI), information not available (N/A).

## 4. Possible Molecular Pathways Mediating AIM2 Activation in IAV

### 4.1. Exogenous DNA

**Figure 2:**
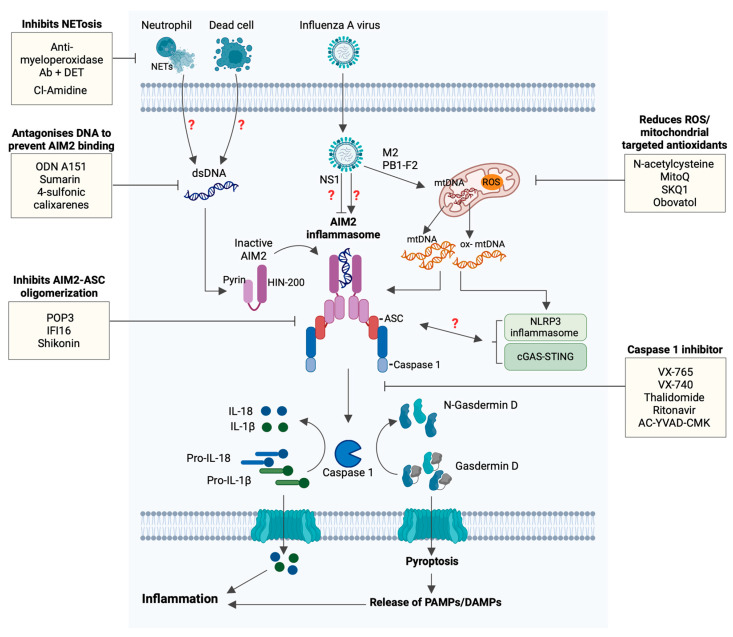
Therapeutic strategies to modulate Absent in Melanoma 2 (AIM2) inflammasome activation in influenza A virus infection. During IAV infection, the AIM2 inflammasome is activated by the detection of cytosolic DNA, such as mitochondrial DNA (mtDNA), which is released following IAV-induced mitochondrial damage by viral proteins M2 and PB1-F2. While the NLRP3 and cGAS-STING pathways are similarly activated by cytosolic mtDNA, the extent of their interaction or redundancy with AIM2 during infection remains undefined, as indicated by a question mark. Viral proteins such as NS1 may antagonise the AIM2 inflammasome as observed during NLRP3 activation and warrants further investigation. Additional sources of cytosolic dsDNA, such as from phagocytosed cells and neutrophil extracellular traps (NETs), could also contribute to AIM2 activation. Upon recognition of DNA by the HIN-200 domain, AIM2 recruits ASC and caspase-1, initiating AIM2 inflammasome assembly and cleavage of pro-IL-1β and pro-IL-18 into their bioactive forms. Caspase-1 also cleaves gasdermin D, a pore-forming protein that induces a lytic cell death called pyroptosis. The release of these pro-inflammatory cytokines, as well as PAMPs and DAMPs, directly contributes to inflammation and its pathogenesis during severe IAV. Potential therapeutic interventions could target various stages of the AIM2 activation pathway. Several agents, Anti-myeloperoxidase Ab + DETC and Cl-Amidine have been identified to inhibit NETosis-induced dsDNA release. N-acetylcysteine, MitoQ, SKQ1, and Obovatol are effective antioxidants that reduce mitochondrial reactive oxygen species (ROS) production. AIM2 antagonists such as ODN-A1511, Sumarin, and 4-sulfonic calixarenes block DNA sensing, while POP3, IFI16 and Shikonin disrupt ASC oligomerisation, thereby preventing AIM2 inflammasome complex formation. Furthermore, VX-765, VX-740, Thalidomide, Ritonavir and AS-YVAD-CMK prevent cleavage and activation of caspase-1 and, in turn, subsequent release of downstream pro-inflammatory cytokines.

Additional studies have provided mechanistic insights into host DNA sources, which may act as ligands for AIM2 during IAV infection (Figure 2). Indeed, subcellular structures such as the mitochondria (mt) contain copies of mtDNA that encode essential elements required for oxidative metabolism. Release and cytosolic localisation of such self-DNA has been observed during mitochondrial dysfunction in viral disease [67,68,69,70,71,72,73,74]. The influenza M2 protein, has recently been shown to disrupt mitochondrial membrane integrity, leading to the release of mtDNA into the cytosol [70]. Further studies have introduced a role for IAV PB1-F2 protein, which, in conjugation with matrix protein 2 (M2), triggers IAV-induced oxidative stress and Tom40-channel-dependent release of oxidised mtDNA into the cytosol [70]. This subsequently stimulated AIM2 inflammasome caspase-1 mediated IL-1β and IL-18 secretion. Indeed, mtDNA has been shown to successfully engage AIM2 inflammasome activation and is positively correlated with disease severity in multiple chronic inflammatory diseases [75,76,77]. Validation of mtDNA-dependent AIM2-inflammasome activation in more physiologically relevant IAV cell lines will be important moving forward, as murine J774A.1 cells and BMDMs are not major cell types infected during IAV. Moreover, it is possible this inflammatory cascade is further amplified by the induction of other inflammasomes like NLRP3 which Moriyama and colleagues have demonstrated mtDNA are also ligands for.

It has been speculated that macrophage-mediated phagocytic uptake of dead and neighbouring damaged cells could indirectly trigger AIM2 (Figure 2). Specifically, lysosomal dysfunction, characterised by impaired membrane permeability, could lead to the release of genomic DNA from apoptotic bodies into the cytosol. This could, therefore, provide ample opportunity for AIM2 to detect and respond. While impaired clearance of dying cells has been observed in systemic lupus erythematosus [78,79], no study has shown a direct link between these ligands and AIM2 during IAV infection. Another possibility is the release of neutrophil extracellular traps (NETs), composed of dsDNA and antimicrobial proteins, which are released by neutrophils during infection. Indeed, NETs have been implicated in the pathogenesis of severe IAV infections [80,81,82,83]. A 2021 study demonstrated that extracellular NETs were phagocytosed by alveolar macrophages and sensed by AIM2 in an LPS-induced ARDS model [84]. The precise mechanisms by which AIM2 detects the extracellular DNA backbone of NETs remain unclear. However, collectively, these findings support mechanisms in which host-DNA may provoke the AIM2 inflammasome during IAV and potentially exacerbate inflammation during IAV. This underscores the need for further research to better understand the repertoire of PAMPs/DAMPs that trigger AIM2 during infection.

### 4.2. Cross-Talk between DNA and RNA Sensors

Evidence suggests there is significant cross-talk that exists between cytosolic DNA sensors that contribute to antiviral immunity. In the context of IAV, it is important to understand the biological intercommunication between them and how they act to amplify or antagonise DNA-induced inflammatory cascades during severe disease. These may result in various downstream responses, including the secretion of IL-1β and IL-18, type I IFNs, TNF cytokines, and pyroptosis.

Notably, like AIM2, the cyclic GMP–AMP synthase (cGAS)–stimulator of interferon genes (STING) pathway is well acknowledged to respond to cytoplasmic DNA; however, it occurs in a caspase-1 independent manner [43,85,86]. Mechanistically, upon successful engagement, cGAS synthesises the secondary messenger c-GAMP, which recruits and oligomerises STING to secrete type I IFNs [85,86]. Secretion of IFNs occurs via STING-dependent interferon-regulatory factor(IRF)3 and TANK-binding kinase 1 (TBK1) activation [87]. Recent findings have suggested that 2′3′ cyclic GMP–AMP (cGAMP) is a positive regulator of AIM2. In MCMV-infected BMDMs, IL-1β was reduced in *cGAMP*^−/−^ but not in WT cells [88]. Though it is possible that multiple PRRs may induce IL-1β during MCMV, microscopy experiments by Swanson and colleagues demonstrated colocalisation of cGAMP with AIM2 and ASC. Noteworthy, NLRP3 was also found to colocalise with cGAMP. In another study, oxidised mtDNA induced both AIM2 and NLRP3 activation during IAV infection [66]. Whilst the relative inflammasome contributions were not investigated, these studies suggest a degree of redundancy exists in sensors capable of activating inflammasomes. The precise manner by which AIM2, NLRP3, cGAS, and other nucleic acid sensors are regulated and function in unison is poorly understood.

Emerging evidence has supported the simultaneous activation of multiple inflammasomes by PAMPs and DAMPs during infection, revealing the complex interplay of innate immune responses. Z-DNA binding protein 1 (ZBP-1), initially characterised as a cytosolic Z-DNA sensor, has since been recognised as a critical detector of Z-RNAs produced by replicating IAV [89,90]. More recently, studies have established a functional role of ZBP-1 as an upstream regulator of NLRP3, where it senses IAV-derived Z-RNAs and promotes the formation of the ZBP1-NLRP3 inflammasome [91,92,93]. This complex induces IAV-inflammatory cell death through a receptor-interacting serine/threonine-protein kinase (RIPK)1-RIPK3-Caspase-8 pathway, a distinct web of apoptotic-necroptotic-pyroptotic mediated cell death [93]. Notably, AIM2 has been shown to be dispensable for ZBP-1 activity during IAV, as *Aim2*^−/−^ BMDMs did not affect ZBP-1 expression [94]. However, BMDMs lacking ZBP-1 completely abrogated caspase-1 activation and almost eliminated IL-1β and IL-18 secretion [93]. Another study found that ZBP-1 was required for IL-1β release during IAV infection [95]. These findings suggest that some interaction between ZBP-1 or downstream components and AIM2 occurs during IAV infection.

### 4.3. Viral Proteins

Previous studies have also established IAV as a potent inducer of IFNs at various stages of infection [96]. The non-structural protein 1 (NS1) of IAV, a multifunctional protein expressed abundantly in infected cells, has been shown to regulate type I IFN transcription, inflammatory responses, and cell death during infection [97]. Mechanistically, it is thought to accomplish this by disrupting RIG-I dependent signalling, cGAS-STING activation, and the ZBP1-NLRP3 inflammasome by sequestering mtDNA or viral RNA during replication, thereby preventing transcription of IFN genes such as IRF3 and IFNβ [70,92,98,99,100]. Indeed, *Mycobacterium tuberculosis*-induced inhibition of IFNβ has been shown to dampen AIM2 activation [101]. Considering IFNs are positive regulators of ISGs, like AIM2, whether NS1 protein also acts as a direct negative regulator of AIM2 to impact the antiviral response should be determined. It is unclear whether AIM2 activation occurs independently, cooperatively, synergistically, or redundantly with other cytoplasmic receptors during IAV infection. Further, the importance of viral proteins and whether these interactions contribute to severe infection outcomes warrants investigation. Defining the regulatory roles and feedback cycles of these sensors is necessary to better understand the multifaceted innate immune response to IAV.

## 5. Therapeutic Potential of AIM2 in Influenza

While the activation of AIM2 is essential for host defence against a plethora of pathogens, dysregulated AIM2 inflammasome activation contributes to the pathogenesis of the disease [39,40,41,42,49,50,51,59,60,61]. The exacerbated inflammatory response to IAV is driven by the activation of inflammasomes, leading to the release of IL-1 family cytokines and induction of pyroptosis [26,27]. Inhibiting AIM2, or upstream and downstream regulators that modulate AIM2 activity, may provide benefit to severe IAV patients (Figure 2). However, given the apparent dual role of AIM2 in promoting antiviral immunity and contributing to inflammation, therapeutic strategies targeting this pathway must be carefully evaluated to ensure protective antiviral responses are not compromised.

### 5.1. Targeting Components of the AIM2 Inflammasome

Selective inflammasome inhibitors have gained significant attention in recent years as a potential therapy for inflammatory diseases, with a major emphasis on the NLRP3 inflammasome. To date, there is no specific pharmacological inhibitor of AIM2 that has been approved for clinical use. ODN A151, a synthetic suppressive oligodeoxynucleotide, antagonises cytoplasmic dsDNA by competitively binding to AIM2 [102]. Structurally, it is composed of four repeats of TTAGGG, which have been found to suppress innate immune responses [102,103]. ODN A151 mediates these effects by preventing ASC recruitment, IL-1β and IL-18 processing, and pyroptosis in BMDMs and bone marrow dendritic cells (BMDCs) [102]. Endogenous PYRIN-only protein 3 (POP3) inhibits DNA-induced AIM2 inflammation activation by competing with ASC [104]. Mouse p202 and recently identified human homolog IFI16-b binds to the AIM2 HIN-domain to inhibit engagement with ASC [46,105,106]. Recently, suramin, a clinically approved drug for African sleeping sickness and river blindness, and its biochemically similar compound, 4-sulfonic calixarenes, were demonstrated as inhibitors by antagonising the HIN-domain dsDNA binding site of AIM2 [107]. They were also found to be effective at inhibiting cGAS-STING and TLR9 at higher concentrations without inducing cytotoxicity [107,108]. However, the therapeutic potential of these compounds in IAV is yet to be explored.

### 5.2. Targeting Release of Self-DNA

Strategies to prevent the release of mtDNA and NETs could serve to limit the activation of AIM2 and subsequent inflammation. Anti-myeloperoxidase antibodies in conjunction with the superoxide dismutase inhibitor (DETC) and PAD4 inhibitor Cl-amidine have been shown to inhibit NETosis during severe disease models such as lupus, sepsis and multiple sclerosis (MS) [80,109,110,111]. N-acetylcysteine (NAC) is believed to exert antioxidant effects by reducing the production of ROS to indirectly inhibit NETosis [112,113]. Flora and colleagues evaluated NAC treatment on patients experiencing IAV or influenza-like symptoms and observed reduced symptom severity towards the A/Singapore/6/86 H1N1 strain [114]. Other mitochondrial-targeted antioxidants, including MitoQ, SKQ1, and Obovatol, act to neutralise mitochondrial ROS, protect glutathione activity, and prevent mitochondrial oxidative damage and thus mtDNA release [115,116]. Additionally, since the cGAS-STING pathway is also activated by mtDNA, ODN A151 also antagonises cGAMP [117]. Therapeutic targeting may have the potential to prevent hyperactivation and the cGAS-AIM2 feedback loop from perpetuating inflammation during IAV. However, the complexity of mitochondrial signalling pathways during IAV and the need for specificity in upstream targeting AIM2 remain challenges that require further exploration.

### 5.3. Targeting Caspase-1

The therapeutic potential of targeting caspase-1 has been largely explored; however, this strategy would also alter responses downstream of NLRP3 and result in increased susceptibility to infections. While multiple synthetic inhibitors have been designed, few have progressed due to low efficacy, toxicity, or non-specific binding. VX-765 and VX-740 had potent and highly selective caspase-1 inhibitory activity in vivo, yet despite this did not successfully complete phase II clinical trials due to toxicity stemming from an incomplete understanding of caspases in disease [118,119,120]. Agreeingly, thalidomide has also demonstrated a significant ability to inhibit caspase-1; however, its clinical use is limited due to its associated teratogenic effects [121]. Studies have shown that Ritonavir, known for its use as a protease inhibitor in HIV, suppresses caspase-1 activity [122]. AC-YVAD-CMK is a well-known inhibitor of caspase-1, resulting in attenuated IL-1β and IL-18 secretion, and more recently has been shown to inhibit NLRP1 inflammasome induced pyroptosis during sepsis-associated acute kidney injury [123]. This has been attributed to combinational activity involving increased release of antioxidants, decreased ROS products, and reduced expression of NLRP1 inflammasome. Furthermore, Shikonin, a plant extract commonly used in Chinese medicine and NF-kB inhibitor, suppressed AIM2 and NLRP3 activation by its direct modulation of ASC oligomerisation and caspase-1 maturation [124].

## 6. Conclusions and Future Directions

The well-established role of AIM2 as a sensor for dsDNA has traditionally framed its activity within the context of bacterial and DNA virus recognition. However, its recently identified involvement in RNA viruses like IAV challenges this conventional view and raises critical questions regarding its function in viral immunity. In this review, we highlight several studies that suggest distinct and potentially multifaceted roles of AIM2 during IAV infection and propose mechanisms that may plausibly link AIM2-IAV.

One key conceptual area that warrants deeper exploration is the interplay between, and relative contributions of, AIM2 and NLRP3, both of which form inflammasome complexes and are modulated by various sensors in the context of influenza-induced inflammatory pathogenesis. Additionally, defining the spectrum of ligands recognised by AIM2, how other sensors may negatively or positively regulate AIM2, and the potential of IAV proteins to directly modulate responses remains a critical area for future study. Resolving these uncertainties will advance our understanding of innate immune sensing and facilitate efforts to more precisely target destructive inflammatory cascades without compromising protective antiviral responses.

The possibility that AIM2 inflammasome activation during IAV infection is strain-specific presents an intriguing area for future investigation. While AIM2 activation itself has not been explicitly explored across different strains, distinct strain-specific inflammatory responses have been documented. Comparative analysis between seasonal H3N2 (AH3N2), 2010 swine-origin H3N2 variant (A(H3N2)v), and H3N2 swine-origin virus containing the M gene from 2009 pandemic H1N1 variant (A(H3N2)vpm) have demonstrated that (A(H3N2)vpm) elicits significantly higher pro-inflammatory cytokine release [125,126,127]. Furthermore, the H5N1 IAV strain has been shown to induce a more robust inflammatory response in primary human alveolar and bronchial epithelial cells, as well as in infected ferrets, compared to the H1N1 strain [13]. Understanding whether AIM2 inflammasome activation differs between these pandemic and seasonal IAV strains could provide critical insights into strain-specific immune modulation. Such findings may also help identify new hot targets for pharmacological intervention and establish AIM2 as a potential biomarker for disease severity and prognosis in IAV patients.

More broadly, this RNA-DNA cross-sensing reactivity exhibited by AIM2 underscores a more adaptable and integrated innate immune response than previously appreciated. It expands our understanding of how the immune system responds to invading pathogens and opens new avenues for therapeutic exploration, especially as new inflammasome modulators progress through clinical trials.

## Data Availability

Data are contained within the article.
